# Exosomes: Cell-Derived Nanoplatforms for the Delivery of Cancer Therapeutics

**DOI:** 10.3390/ijms22010014

**Published:** 2020-12-22

**Authors:** Hyosuk Kim, Eun Hye Kim, Gijung Kwak, Sung-Gil Chi, Sun Hwa Kim, Yoosoo Yang

**Affiliations:** 1 Center for Theragnosis, Biomedical Research Institute, Korea Institute of Science and Technology (KIST), Seoul 02792, Korea; hyoseog7@kist.re.kr (H.K.); ehkelly@kist.re.kr (E.H.K.); kwakgijung@gmail.com (G.K.); 2 Department of Life Sciences, Korea University, Seoul 02841, Korea; chi6302@korea.ac.kr; 3 Department of Ophthalmology, Johns Hopkins University School of Medicine, Baltimore, MD 21231, USA

**Keywords:** extracellular vesicle, exosome, cancer therapeutic, drug delivery, exosome engineering

## Abstract

Exosomes are cell-secreted nanovesicles that naturally contain biomolecular cargoes such as lipids, proteins, and nucleic acids. Exosomes mediate intercellular communication, enabling the transfer biological signals from the donor cells to the recipient cells. Recently, exosomes are emerging as promising drug delivery vehicles due to their strong stability in blood circulation, high biocompatibility, low immunogenicity, and natural targeting ability. In particular, exosomes derived from specific types of cells can carry endogenous signaling molecules with therapeutic potential for cancer treatment, thus presenting a significant impact on targeted drug delivery and therapy. Furthermore, exosomes can be engineered to display targeting moieties on their surface or to load additional therapeutic agents. Therefore, a comprehensive understanding of exosome biogenesis and the development of efficient exosome engineering techniques will provide new avenues to establish convincing clinical therapeutic strategies based on exosomes. This review focuses on the therapeutic applications of exosomes derived from various cells and the exosome engineering technologies that enable the accurate delivery of various types of cargoes to target cells for cancer therapy.

## 1. Introduction

**Figure 1:**
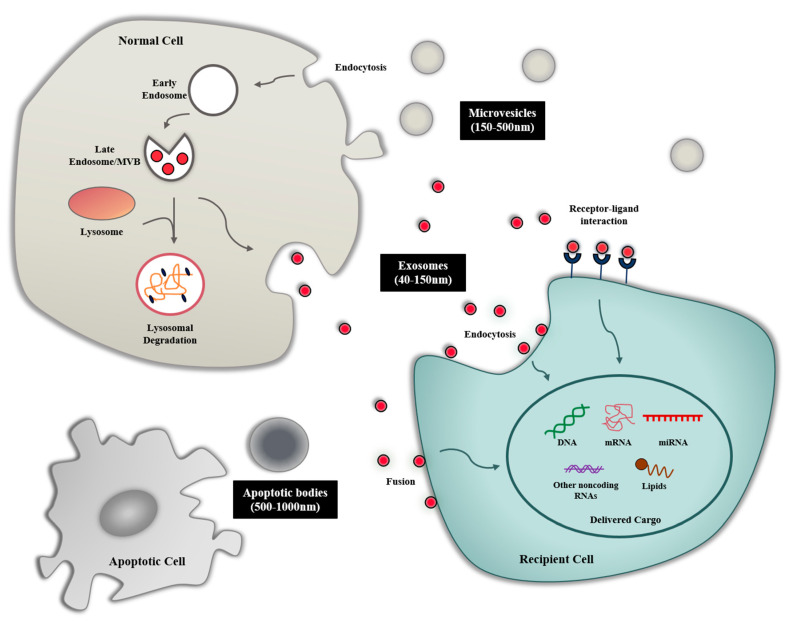
Biogenesis and uptake mechanisms of exosomes. Early endosomes generated through invagination of the plasma membrane form multivesicular bodies (MVB) through inward budding, which releases exosomes through the exocytosis. Released exosomes can be taken up via different mechanisms: endocytosis, membrane fusion, receptor–ligand interaction.

Extracellular vesicles (EV) are one of the means of intercellular communication between adjacent and distant cells. EVs refer to all membrane vesicles that are naturally released from cells and are classified based on their biogenesis pathway, function, size, etc. [1]. EVs are commonly composed of three members: (i) apoptotic bodies released by the cell undergoing programmed cell death, with size ranging from 500 to 1000 nm, (ii) microvesicles emerging from the budding of plasma membranes with a diameter of 150–500 nm, and (iii) exosomes with a diameter of 40–150 nm that are derived from endosomes (Figure 1) [2].

Among them, exosomes play essential roles as communication mediators between cells and contain many important biomolecules such as proteins, nucleic acids, and lipids [3]. These cargoes of exosomes vary depending on the origin of exosomes and their biological state. More recently, exosomes have been known to mediate many physiological and pathological processes by presenting various antigens through the interactions of receptor–ligand between exosomes and the cell membrane, releasing cargo through internalizing into targeted cells, or delivering their surface proteins through membrane fusion [4,5,6]. Due to the discovery of these roles of exosomes, they have recently received the most research attention as a promising drug delivery tool that can overcome the shortcomings of artificial nanoparticles.

The hurdles of some artificial nanoparticles, such as immune recognition [7], inflammatory toxicity [8], and rapid clearance [9], can be overcome due to the biochemical composition of exosomes similar to the cell membrane of exosomes. In contrast to artificial nanoparticles, exosomes have relatively low immune clearance and low cytotoxicity due to their endogenous origin and high biocompatibility. Furthermore, exosomes are capable of delivering both hydrophilic and hydrophobic molecules, and they have efficient target homing abilities to tumor sites that are potentially attributed to their multivalent display of cell-derived surface moieties [10,11].

In this review, we aim to provide a comprehensive understanding of exosomes, including their biogenesis, function, therapeutic applications for cancer treatment, as well as various engineering techniques in drug delivery.

## 2. Biogenesis and Composition of Exosomes

### 2.1. Biogenesis of Exosome

The exosome is a small vesicle of endocytic origin with a diameter of 40–150 nm and contains many types of biomolecules such as proteins and nucleic acids [2]. The biogenesis of exosomes is a complex biological process that has yet to be fully elucidated. First, the biogenesis of exosomes is primarily initiated in the endocytosis process in the lipid raft domain of the plasma membrane. The intracellular early endosomes are generated by the inward budding of the plasma membrane, and these early endosomes become the late endosome with the assistance of the Golgi complex. During this process, intraluminal vesicles (ILVs), which we refer to as exosomes, are formed in the late endosomes through the endosomal sorting complexes required for transport (ESCRT)-dependent and ESCRT-independent mechanisms. Various biomolecules within the cells can be accumulated during the inward budding of the endosomal membrane that form multivesicular bodies (MVBs) [12]. Finally, most of the MVBs fuse with the plasma membrane of the cells, and ILVs are released to extracellular environment by exocytosis. MVBs that are not fused to the plasma membrane are degraded by lysosomes. The process by which MVB docks to the plasma membrane is known to be regulated by Rab GTPase [13]. In addition to ILVs, recent studies have reported that small-sized microvesicles (around 150 nm) budding from the plasma membrane can also be included in exosomes members [14].

### 2.2. Exosome Composition

Even exosomes derived from the same cell are heterogeneous in their size and cargo. However, some cargoes are partially common in exosomes derived from various origins. [15]. According to various exosome studies, exosomes contain numerous biomolecules such as lipids, proteins, RNAs, and DNAs. Most lipids in exosomes are known to be components of the plasma membranes such as cholesterol, sphingomyelin, and phosphatidylserine [16]. The proteome of exosomes include proteins involved in membrane transport or fusion (Rab GTPases, annexins), proteins associated with exosome biogenesis (ESCRT complex, Alix, TSG101), heat shock proteins (HSP70, HSP90), integrins, tetraspanins (CD63, CD81, CD82) [12,16,17], myosin heavy chain (MHC) class II proteins, etc. [18]. In addition, there are many proteins that are expressed on the surface of exosomes and interact with surface receptors of recipient cells to induce intracellular signaling. All RNA species, such as microRNA (miRNA) and messenger RNA (mRNA) as well as transfer RNA and long noncoding RNA, have been identified in exosomes [19,20]. In many studies, it has been reported that specific RNAs are actively, not passively, sorted and carried into exosomes and subsequently affect the transcriptome of recipient cells [3,21]. Interestingly, Batagov et al. also found that the 3’-untransrated region (UTR) of mRNA could enter the exosome via a specific RNA fragment [22]. In addition, Balaj et al. identified single-stranded DNA in the exosome [23], and Kalluri and coworkers provided the evidence that exosomes contain double-stranded genomic DNA fragments of 10 kb or more using whole genome sequencing [24].

## 3. Exosome-Mediated Intercellular Communication

### 3.1. Exosome Uptake

According to current studies, the mechanism by which exosome signals are transferred to recipient cells can be largely defined in three methods: receptor interaction, direct membrane fusion, and endocytosis/phagocytosis. For example, Cosseti et al. found that interferon-γ (IFN-γ) binds to exosome-associated IFN-γ receptor 1 to form a complex, which in turn activates signaling transduction through this complex in neural stem/precursor cells [4]. In addition, some studies have suggested that exosomes transfer their contents to target cells by fusion with a membrane of recipient cells, but the exact molecular mechanism has not been elucidated [6]. Recently, many studies suggest that internalization via endocytic pathways, such as clathrin-mediated endocytosis, lipid raft-mediated endocytosis, macropinocytosis, caveolin-mediated endocytosis, and phagocytosis, is the main method of exosome uptake [5]. These findings suggest that exosome uptake is cell-specific by the interaction of surface molecules between specific cells and exosomes.

### 3.2. Exosome-Mediated Intercellular Communication in Tumor Microenvironment

Exosomes play crucial roles in intercellular communication by delivering biomolecules. Exosome-derived biomolecules secreted by donor cells could effectively change the biological response of recipient cells. Zhang et al. found that the exosome with a significant amount of miRNA-150 secreted by human monocytic leukemia cell line (THP-1) could contribute to target cell migration through the inhibition of target gene expression [25]. In another study, Wang et al. demonstrated that the exosome-mediated delivery of transient receptor potential polycystic 2 (TRPP2) siRNA significantly inhibits TRPP2 expression and the epithelial–mesenchymal transition of FaDu cells [26]. Several studies also proved that exosomes containing functional proteins can mediate cell-to-cell communication and have critical effects on the signaling pathway of the target cell, which is related with cancer progression [27]. Therefore, exosome-mediated cell-to-cell interaction is an effective way to deliver diverse cellular biomolecules and affect functions and phenotypes of target cells.

In our body, natural-killer (NK) cells are responsible for immune surveillance and serve as the first-line defense in regulating cancer growth and metastasis. NK cell-derived exosomes endow cytotoxic activity to tumor cells by carrying killer proteins together with typical NK and exosome markers [28].

Similar to NK cells, macrophages play an important role in innate host defense and in killing tumor cells by producing reactive oxygen/nitrogen species and pro-inflammatory cytokines such as tumor necrosis factor-alpha (TNF-α) and interleukin 6 (IL-6). Macrophages are broadly classified into M1 or M2 types, and tumor-associated macrophages (TAMs) in the tumor microenvironment are known to exhibit an M2-like phenotype. These TAM-derived exosomes induce immune suppression for tumor progression by mediating cell-to-cell communication with other immune cells such as T cells [29].

**Figure 2:**
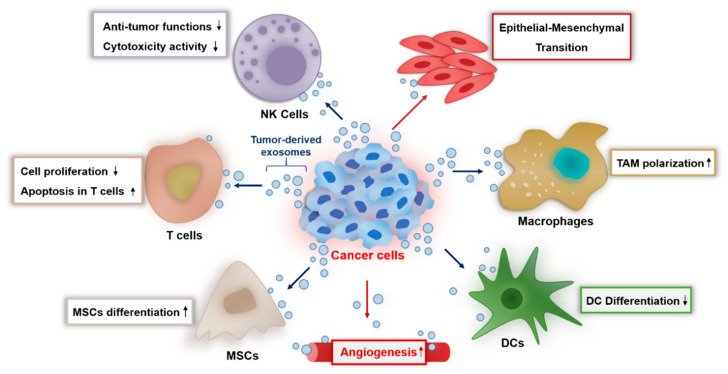
Overview of the role of tumor-derived exosomes in the tumor microenvironment. Exosomes secreted by cancer cells can induce epithelial-mesenchymal transition in other cancer cells and promote the differentiation of mesenchymal stem cells (MSCs). Cancer cells can promote angiogenesis and polarize macrophages to tumor-associated macrophages (TAM), a tumor-supporting phenotype. Tumor-derived exosomes are able to suppress the antitumor response of immune cells such as natural-killer (NK) cells, T cells, and dsendritic cells (DCs).

Tumor-derived exosomes (TEXs) released from malignant cells alter the physiology of macrophages [30], reduce the activity of natural-killer (NK) cells [31], and stimulate tumors by regulating the role of T cells (Figure 2) [32]. TEXs also play an important role in tumor metastasis by regulating the tumor microenvironment (TME) [33,34]. For example, breast cancer cell-derived exosomes deliver certain oncogenic proteins and miRNAs such as miRNA-130a and miRNA-328, which contribute to increase tumor growth and metastasis [35]. Exosomes derived from human pancreatic cells promote metastasis and are critically relevant to the immune-suppressive functions [36]. Similarly, Wang et al. identified that TEXs in gastric tumor cell microenvironment develop primary tumor growth by inducing an immunosuppressive PD1^+^ tumor-associated macrophages population that inhibits CD8^+^ T-cell function [37]. The study of TEXs being involved in angiogenesis has also been widely described. Angiogenesis is the key hallmark of tumor progression, which is directly involved in tumor progression by supplying tumor-associated blood vessels and the target of the cancer immunotherapy. TEXs participate in inducing new vessels at the early stage of cancer development in various tumor types including glioblastoma, malignant mesothelioma, and hypoxic lung cancer [38]. Additionally, the amount of these TEXs is 10 times greater than the exosomes released from normal cells due to alterations in the tumor metabolism.

Therefore, the application of TEX as biomarkers of cancer therapy is becoming increasingly developed due to their function in TME. The role of TEXs as a biomarker has been studied in various recent studies. One study demonstrated that TEXs in non-small cell lung cancer (NSCLC) could also be used as therapeutic biomarkers as potential cancer diagnostic indicators [39]. The other study led by Mousavi et al. investigated that TEXs controlling cancer development could be potential biomarkers of colorectal cancer by detecting exosomes containing tumor-related miRNAs [40].

These findings including various effects of TEXs on cancer pathogenesis support that they may become potential therapeutic agents and tools for cancer therapy.

## 4. Exosomes as Drug Delivery Vehicles

**Figure 3:**
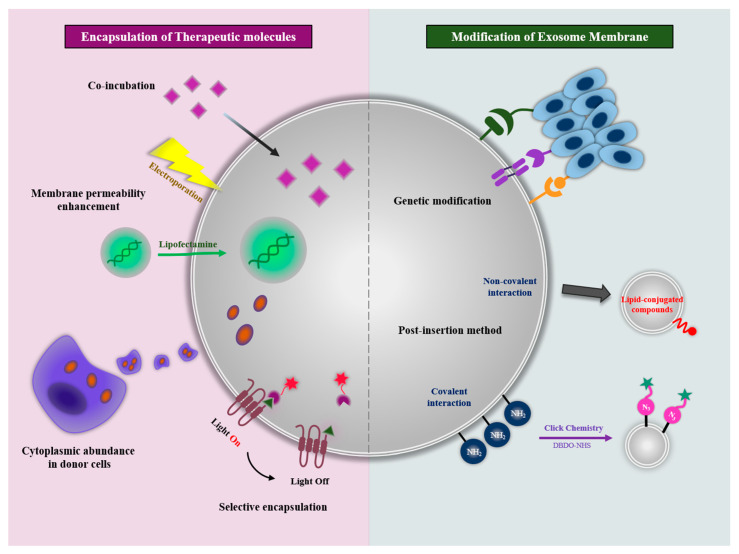
Engineering methods of exosomes as a nanoplatform for improving therapeutic efficacy. Exosomes could be therapeutically engineered with the encapsulation of therapeutic molecules and modification of the exosome membrane. Encapsulation methods involve co-incubation (direct mixing), membrane permeability enhancement (physical/chemical stimuli), cytoplasmic abundance in donor cells, and selective encapsulation via machinery related with exosome biogenesis and release. Membrane modification methods involve genetic modification and the post-insertion method.

As mentioned previously, cell-derived exosomes have shown potential therapeutic effects through cargo contents and functions received from donor cells. This potential can be maximized by engineering the exosome as a natural nanoplatform for drug delivery. With the advancement of nanotechnology, nanomedicine has made an important contribution to improving the loading capacity, biodistribution, and target accumulation of therapeutic molecules [41]. The advances in the field of nanomedicine have been applied to exosomes through the encapsulation of therapeutic molecules and modification of the exosomal membranes (Figure 3).

### 4.1. Encapsulation of Therapeutic Molecules

Recently, many papers have reported the loading of a variety of therapeutic molecules including chemical drugs, proteins, and nucleic acids into exosomes. As a nanoplatform, the inner space of exosomes can be the room for drug loading. In addition, as previously mentioned, exosomes have internal contents, including proteins and miRNAs, inherited from donor cells. The use of exosomes as a drug delivery vehicle can be mainly discussed in four ways: (1) co-incubation, (2) membrane permeability enhancement, (3) cytoplasmic abundance in donor cells, and (4) selective encapsulation in exosome.

#### 4.1.1. Co-Incubation

Co-incubation with exosomes and therapeutic molecules is the simplest encapsulation method using the space of the membrane lipid bilayer. The small hydrophobic molecules such as curcumin [42] and doxorubicin [43] were able to be loaded by incubation with exosomes for 5 min and 1 h in RT and 37 °C, respectively. The incorporation of hydrophobic drugs into exosomes increased the solubility and stability of the hydrophobic drugs in blood circulation. In addition, the accumulation of drug-loaded exosomes at brain tumors was observed across the blood–brain barrier through the biodistribution change of hydrophobic drugs. However, the molecules that can be loaded into exosomes by co-incubation are limited to hydrophobic small molecules that interact with the exosomal membranes. Molecules with high molecular weight, such as proteins and nucleic acids, are difficult to be loaded into exosomes by this way. In addition, even small hydrophobic molecules cannot fully utilize the internal capacity of exosomes with co-incubation methods. Therefore, various drug encapsulation methods have been developed to increase the loading capacity regardless of the chemical properties and molecular weight of the therapeutic molecule.

#### 4.1.2. Membrane Permeability Enhancement

To overcome the limitations of the co-incubation method, new approaches have been proposed to improve exosomal membrane permeability through physical or chemical stimulation. These stimulations are commonly used methods to improve intracellular uptake or induce the membrane deformation of cells, and they can also be applied to exosomes with the same membrane structure. Membrane permeability can be improved by the generation of pores on the exosomal membranes, disruption of membrane integrity, or transfection with positively charged agents. While these approaches ideally increase drug loading efficiency, the aggregation of exosomal membranes can be induced by these stimulations.

As pore generating stimuli, electroporation and saponin permeabilizer have been studied for membrane permeability enhancement. Electroporation is a method of incubating exosomes with the desired therapeutic agents and exposing them to a repetitive electric field of a certain voltage to make a pore in the exosomal membranes to encapsulate therapeutic agents [44]. This method has been applied preferentially when incorporating nucleic acids such as siRNA, mRNA, and miRNA into exosomes, and it showed efficient encapsulation, but a size-dependent limitation was still observed in the electroporation of large linear DNAs [45]. As another method, a saponin permeabilizer is a surfactant that can make pores via cholesterol removing without ruining the cellular membranes [46]. Fuhrmann et al. reported the encapsulation of porphyrins, a cytotoxic drug for photodynamic therapy, into exosomes using saponin permeabilizer [47]. Saponin-mediated encapsulation increased the drug-loading efficiency compared to the methods for the co-incubation, electroporation, and extrusion. However, since saponin has hemolytic activity [48] and is difficult to remove from exosomes, the amount of saponin used for drug loading should be controlled for in vivo applications.

To disrupt exosomal membrane integrity, sonication has been explored for drug encapsulation into exosomes. The membrane deformation through the ultrasonic homogenizer probe causes the drugs to diffuse into exosomes. Kim et al. demonstrated that exosomal membrane microviscosity was decreased by sonication [49]. As the size of exosomes increased, sonication loaded a higher concentration of paclitaxel within exosomes compared with other methods, including co-incubation and electroporation. These paclitaxel-loaded exosomes showed effective antitumor activity in a metastatic lung cancer mouse model. Another exosomal membrane disruption method for drug loading is the extrusion. During extrusion, the exosomes undergo mechanical stress while passing through the narrow pores, and their membranes are disrupted and vigorously mixed with the drugs. In addition, Haney et al. reported the catalase encapsulation in exosomes using extrusion [50]. The catalases and exosomes were extruded 10 times with 200 nm pores. With this extrusion condition, catalase-loaded exosomes exhibited higher drug-loading efficiency compared with the co-incubation method. Similar to sonication, exosome size was increased after extrusion. However, accurate understanding about how to change the membrane structure and property is still insufficient.

Lastly, therapeutic nucleic acids can be loaded into the exosomes with transfection agents. Shyam et al. showed siRNA encapsulation in exosomes using a commercial transfection agent, lipofectamine [51]. The RAD51 siRNAs were mixed with lipofectamine at RT for 10 min; then, the exosomes were added into the mixture and incubated at RT for 30 min. After purification, the siRNA-loaded exosomes showed siRNA transfer to recipient cells and induced therapeutic effects. However, the method using the lipofectamine exhibited low drug loading efficiency compared with the electroporation method.

#### 4.1.3. Cytoplasmic Abundance in Donor Cells

One of the main approaches for drug encapsulation into exosomes is the overexpression of the drug in donor cells. Therapeutic molecules including chemicals, nucleic acids, proteins, and nanoparticles in the cytoplasm can be loaded in the exosomes due to their abundance. Pascucci et al. treated a low dose of paclitaxel to mesenchymal stroma cells for 24 h [52]. After subculture, the paclitaxel-loaded exosomes were obtained from the mesenchymal stroma cells. These exosomes inhibited proliferation of CFPAC-1 human pancreatic cells in vitro. Zhang et al. transfected siRNA for hepatocyte growth factor (HGF) to human embryonic kidney 293T (HEK293T) cells [53]. The HGF siRNA were contained in isolated exosomes and transferred to the SGC-7901 cancer cells. Furthermore, exosomes loaded with HGF siRNA showed antitumor effects via HGF silencing in vivo by intravenous tail injections. Therapeutic nanoparticles also can be loaded into exosomes. Silva et al. investigated combining magnetic nanoparticles with therapeutic drugs [54]. The iron oxide nanoparticles and drugs ingested by THP-1 macrophage cells were successfully loaded into the exosomes. The loaded exosomes were magnetically responsive and manipulated by magnetic forces.

Collectively, the method inducing the cytoplasmic abundance in donor cells showed a facile encapsulation of various therapeutic molecules with a simple mechanism. However, note that the toxicity of donor cells and inefficient drug loading limit its broad applications.

#### 4.1.4. Selective Encapsulation in Exosome

To overcome the limitations of the method for cytoplasmic abundance in donor cells, a method of selective drug encapsulation into donor cells was explored. Particularly, unlike chemical drugs, large molecules including proteins and nucleic acids are hardly passively encapsulated into the exosome lumen. However, therapeutic molecules can be selectively loaded into exosomes by targeting the constituent proteins of exosomes involved in exosome biogenesis and packaging (e.g., ESCRT machinery). The interaction between the constituent protein of the exosome and the therapeutic molecule leads to a specific encapsulation of the molecule into the exosome.

Di Bonito reported a fusion protein which is the Nef exosome-anchoring protein fused with human papillomavirus E7 (Nef/E7) for exosome-based immunization. [55]. The Nef/E7 fusion protein was successfully loaded into exosomes with Nef specific manner. The Nef/E7-loaded exosomes induced an E7-specific immune response in vivo. Another target for selective encapsulation is ESCRT machinery. In a similar approach, targeting chicken egg ovalbumin (OVA) to exosomes was achieved by fusing it to the C1C2 domain of lactadherin that has an intrinsic property to bind to lipid membranes [56]. De Gassart et al. made a chimeric protein containing the cytosolic domain of the viral Env protein and the ectodomain of CD8 (CDTM-BLVeCD8) [57]. The CDTM-BLVeCD8 was found to be very efficient in exosomes via peptide motifs potentially recognized by components of the ESCRT machinery in K562 cells. In addition, as a light triggering an active encapsulation method, Yim et al. reported the exosomes for protein loading via optically reversible protein–protein interactions (EXPLORs) [58]. The reversible interaction was conducted between a photoreceptor cryptochrome 2 (CRY2) and CRY-interacting basic-helix-loop-helix (CIBN) by blue-light illumination. Interaction between a CRY2-cargo protein and a CIBN-CD9 protein induced the cargo loading into exosomes with light. The encapsulation method using the constitutive proteins of exosomes showed an efficient selective loading of the therapeutic molecules into exosomes.

For the selective encapsulation of nucleic acids into exosomes, exosome-enriched RNAs (eRNAs) have been studied with the distinguishing of enriched sequences in the exosomes [59]. The eRNAs share three specific sequence motifs including ACCAGCCU, CAGUGAGC, and UAAUCCCA that may function as cis-acting elements targeting to exosomes. These results aid in our understanding of the selective targeting of candidate RNAs for therapeutic purposes. In addition, tumor-suppressor miRNAs present in tumor-derived exosomes were investigated. For example, Teng et al. reported the major vault protein (MVP)-mediated selective sorting of tumor suppressor miRNA, miR-193a [60]. The knockout of MVP failed to sort miR-193a into exosome and caused miRNA accumulation in the donor cancer cells. The key elements of the RNA-induced silencing complex (RISC), GW182 and AGO2, are known to be related to the MVBs and may be involved in the distribution of miRNAs to exosomes [3,61]. In addition, the depletion of Myoferlin in cancer-derived exosomes was shown to have functionally reduced capacity to transfer nucleic acids to human endothelial cells [62].

These proteins such as MVP, GW182, AGO2, and Myoferlin may be be a tool for loading specific nucleic acids into exosomes. More detailed and accurate bioinformatics analysis will be needed to discover new targets for encapsulation into exosomes.

Although exosomes have promising therapeutic potential, there are several challenges that must be overcome before transitioning from the laboratory stage to clinical use. Some of these challenges include the low yield of exosomes and non-standardized isolation and purification methods. In order to overcome these hurdles, biomimetic exosomes that can be generated in all cell types with characteristics similar to exosomes for an alternative therapeutic drug modality have been developed and studied (Table 1).

### 4.2. Modification of Exosomal Membranes

The exosomal membranes, the surface of the nanoplatform, is exposed to the external environment. In the field of nanomedicine, surfaces play an important role in therapeutic applications including extended circulation and specific activity targeting. Exosomes have the same composition and formation as the donor cell membrane. Thus, they express an intrinsic membrane pattern that includes lipid composition, adhesion molecules, specific ligands, and membrane-associated enzymes. The exosome engineering with modification of their membrane mainly focused on adjusting this pattern to increase the therapeutic effect.

Exosomal membranes can be modified with membrane proteins expressed by transfection with plasmid DNAs or mRNAs in donor cells. Since membrane proteins on the cellular membranes are inherited to exosomes with the native forms associated with lipid bilayers, the exosome is a unique platform for maximizing the membrane protein activity [72]. With membrane modification through the expression of functional membrane proteins, engineered exosomes could have a new targeting ability and exhibit the extension of circulation time. For example, it has been reported the HEK293T cells-derived exosomes expressing the exosomal protein Lamp2b fused with interleukin 3 (IL3). The exosomes were able to selectively target Chronic Myeloid Leukemia cells that overexpress IL3 receptors [73]. Lamp2b was also used to specific delivery therapeutic exosomes to the mouse brain. Targeting was achieved by engineering the dendritic cell-derived exosomes to express Lamp2b fused to the neuron-specific RVG peptide [74]. Another study showed that the signal-regulatory protein alpha (SIRPα) modified exosomes for blocking the “don’t eat me” signal via targeting CD47 on tumor cells [75]. For the effective expression of SIRPα proteins on exosomal membranes, the SIRPα ectodomain was inserted in pDisplay vector. The engineered exosomes expressing SIRPα was able to antagonize CD47 on tumor cells in vitro, and they successively accumulated at tumor sites, inhibiting tumor growth in vivo. Since the transmembrane domain of platelet-derived growth factor receptor-beta (PDGFR-β) of the pDisplay vector has a property to dimerize by itself at lipid raft domains, SIRPα proteins could induce efficient CD47 blockade with higher affinity compared with monomeric SIRPα when expressed on the exosomal surface. The SIRPα–CD47 interaction also was used for the extension of blood circulation time of exosomes. Kamerkar et al. suggested that CD47-modified exosomes were protected from phagocytosis by monocytes and macrophage [76]. The exosomes with high levels of CD47 expression showed higher retention in the circulation, resulting in enhanced RNAi delivery to specifically target oncogenic KRAS in pancreatic tumors.

Alternatively, rather than using molecular cell biology approaches to enable downstream modifications of exosome surface macromolecules, exosomal membranes can be modified by post-insertion methods. The post-isolation modification techniques enable the functionalization of exosome surfaces with specific moieties to improve targeting ability and biodistribution and allow tracking in vivo and in vitro. The exosome surface can be essentially modified by (i) covalent interaction between functionalizing molecules or the chemical linker and the amine groups, which are reactive functional units widely expressed on exosomes’ surfaces, and (ii) non-covalent interaction based on lipid-conjugated compounds post-insertion into exosomal membranes. Recent study has reported that polyethylene glycol (PEG)-modified exosomes were able to avoid opsonization and extend the circulation half-life of exosomes. These exosomes were modified through the incorporation of 1,2-distearoyl-sn-glycero-3-phosphoethanolamine into the lipid layer of the exosome. Furthermore, for targeting lymph nodes, the PEG’s distal end was functionalized with amine groups followed by conjugating with mannose-isothiocyanate [77]. Kim et al. also reported the PEGylated exosomes having a targeting moiety [78]. They developed the aminoethyl anisamide-polyethylene glycol (AA-PEG) vector to target the sigma receptor overexpressed by lung cancer cells. The AA-PEG vector had the 1,2-distearoyl-sn-glycero-3-phosphoethanolamine for the interaction with the exosomal membranes. The exosomes derived from bone marrow-derived macrophages mixed with AA-PEG vector and showed accumulation at cancers with systemic administration. Other post-isolation functionalization techniques can utilize specific receptors or antigens. For example, as the A33 antigen has been proven to be overexpressed in colorectal cancer cells, exosomes isolated from these cells present the A33 antigen on their surface. These exosomes are loaded with doxorubicin and functionalized with superparamagnetic iron oxide nanoparticles coated with high-density A33 antibodies, showing antitumor activity toward colorectal cancer with reduced systemic toxicity [79].

In this way, the development of exosomal membrane engineering technology can maximize the efficacy of exosomes through an improved circulation time and targeting ability of exosomes.

## 5. Therapeutic Applications of Exosomes for Cancer Therapy

With advantages such as high biocompatibility, increased circulating stability, low immunogenicity, and toxicity, exosomes can be used as attractive therapeutic delivery vehicles for delivering functional genetic material to the targeted cells. A number of studies have been conducted to show that a variety of cell-derived exosomes can be used as therapeutic agents for cancer therapy (Table 2).

### 5.1. Tumor-Derived Exosomes

TEXs are commonly used with chemotherapeutics to enhance the efficacy of cancer treatment because it is easy to deliver to tumor cells through the surface proteins of TEX. TEXs loaded with doxorubicin through electroporation showed a considerable inhibition of tumor growth than the free doxorubicin group and also showed the cardioprotective effect by restricting the uptake to myocardial endothelial cells [63].

Tumor cells express antigens that can be recognized by cytotoxic T lymphocytes (CTLs). TEXs are also able to present tumor antigens, indicating that TEXs can be modified for cancer immunotherapy. Previous studies have shown that dendritic cells (DCs) can take up TEXs containing donor antigens and induce antigen-specific CTL responses in vitro or in vivo. As a result of the stronger CTL activation and faster DC uptake rate, the tumor-suppression effect of TEX-loaded DCs was significantly higher than that of tumor lysate-loaded DCs in a myeloid leukemia WEHI-3B-bearing mouse model [80]. Meanwhile, TEXs are also used in immune suppressors that efficiently regulate tumor-related immune response through modulating the signaling pathway, maintaining the effects of regulatory T cells and processing enzymatic activity [81]. Rong et al. found that TEXs in breast cancer act as critical immune suppressors by carrying transforming growth factor-β, which is a potent immunosuppressive factor on T cell expression [82].

Recent research has confirmed that the M2 isoform of pyruvate kinase plays a key role in catalyzing glycolysis. Circular RNA identified as a sponge of miR-122 targeting PKM2 was loaded onto TEXs and delivered to drug-resistant colorectal cancer. TEXs inhibited glycolysis by reprogramming tumor metabolism, weakened drug resistance, and showed excellent anticancer effects in in vitro and in vitro experiments [83]. TEX, which is also involved in the progression and metastasis of cancer, has been extensively studied as a biomarker for the diagnosis of cancer and for monitoring the formation of atherosclerosis, but targeting a specific function of TEX could also be a promising therapeutic approach for preventing or delaying cancer recurrence.

**Table 2:** Summary of studies using exosomes for cancer therapy.

Source of Exosome	Strategy	Outcome	Target Cancer Type	References
Tumor cell-derived exosome				
Human breast cancer (MDA-MB-231)	Loading with doxorubicin through electroporation	Inhibition of tumor progression and enhancing the cytotoxicity of doxorubicin	Breast	[63]
Human breast cancer (MDA-MB-231 and BT-474)	Carrying transforming growth factor-β (TGF-β) and interleukin-10 (IL-10) which exhibit the immune-regulatory functions, a potent immunosuppressive factor on T cell expression	Providing biomarkers for cancer diagnosis	Breast	[82]
Human glioblastoma (A172)	Carrying angiogenic proteins and RNA that induce new vessels at the early stage of cancer development	Providing biomarkers for cancer diagnosis	Glioblastoma	[38]
Non-small cell lung cancer (from NSCLC patients)	Carrying exosomal proteins such as alpha-2-HS-glycoprotein (AHSG) and extracellular matrix protein 1 (ECM1)	Providing biomarkers for cancer diagnosis	Non-small cell lung cancer (NSCLC)	[39]
Colorectal cancer	Increasing the transfer of small molecules including growth factors, chemokines, and RNAs	Providing biomarkers for cancer diagnosis	Colorectal cancer	[40]
**Stem cell-derived exosome**				
Mouse bone marrow MSCs (BM-MSCs)	Carrying MiR-16, which downregulates the expression of VEGF in the TME	Inhibition of angiogenesis	Breast cancer (4T1)	[84]
Human MSCs	Carrying MiR-100, which downregulates the expression of VEGF by modulating mTOR/HIF-1α signaling	Inhibition of angiogenesis	Breast cancer (MCF-7 and MDA-MB-231)	[85]
Human MSCs	Carrying MiR-124, which induces S-phase arrest through the downregulation of other CDKs	Inhibition of proliferation	Ovarian cancer	[86]
Immune cell-derived exosome				
Dendritic cell (DC)	Activating NK cells and T cells and inducing the secretion of interferon-γ (IFN-γ)	Inhibition of tumor progression	Melanoma (B16), Colon adenocarcinoma (MC38), Squamous cell carcinoma (KLN205)	[87,88,89]
			Non-small cell lung cancer (NSCLC)	[90]
	Loading factors that stimulate a wide range of immune cells to enhance antigen-specific T cell responses	Inhibition of tumor progression	Melanoma (B16/OVA)	[91]
	Enhancing antitumor immunity through TRL3 stimulation during the maturation of bone marrow derived DCs	Improve antitumor immunity and application in therapeutic cancer vaccines	Melanoma (B16F10)	[92]
	Expressing hepatocellular carcinoma antigen α-fetoprotein through lentivirus transfection	Inhibition of tumor progression	Hepatocellular carcinoma	[93]
Natural-killer cell (NK)	Increasing the proliferation rate of NK cells involving FasL and perforin	Improve antitumor immunity	Melanoma	[94]
	Treatment with dextran sulfate which block scavenger receptor A and preventing the ingestion of exosomes in the liver	Inhibition of tumor progression and improve antitumor immunity	Glioblastoma	[95]
	Activation of human NK cells with artificial antigen-presenting cells	Inhibition of proliferation	Acute lymphoblastic leukemia (SupB15, NALM-6), neuroblastoma (CHLA-136, CHLA-255), and breast cancer (MCF7)	[96]
CD8 + T cells	Inducing the apoptosis of mesenchymal stem cells	Inhibition of tumor progression	CMS5a, CMS5m, CMS7, CT26, 4T1, B16 and B16F10	[97]
Macrophages	Enhancing a pro-inflammatory cytokine, which induces the cytotoxic T cell immune response	Improve antitumor immunity and application in therapeutic cancer vaccines	Melanoma (B16F10)	[98]
	Loading paclitaxel and doxorubicin through various methods to overcome multidrug resistance in MDCKMDR1 (Pgp+) cells	Enhancing the cytotoxicity of paclitaxel and doxorubicin	Murine Lewis lung carcinoma cell subline (3LL-M27)	[49]
Other cells-derived exosome				
Human embryonic kidney 293T (HEK293T)	Carrying Imatinib or BCR-ABL siRNA which express IL-3	Inhibition of tumor progression	Chronic myelogenous leukemia (LAMA84, K562)	[73]
	Carrying super-repressor IκB (srIκB) to a therapeutic target	Inhibition of inflammatory responses	Monocytic THP-1 cells and human umbilical vein endothelial cells	[99]
	Carrying therapeutic GPI-anchored hyaluronidase to the overly accumulated ECM	Inhibition of tumor progression and activation of infiltration of T cells	Prostate cancer cell (PC3)	[100]
	Loading with doxorubicin through electroporation	Inhibition of tumor progression	Primary pulmonary artery smooth muscle cells	[101]
	Carrying MiR-497 which suppress cell proliferation, migration and angiogenesis of tumors	Inhibition of tumor progression	Non-small cell lung cancer (NSCLC)	[102]
Adipocyte	Alleviating lung cancer metastasis by activating MMP9	Promoting cancer cell invasion and metastasis	Lung cancer (3LL)	[103]
	Inducing a metabolic reprogramming in tumor cell	Promoting cancer cell invasion and migration	Melanoma	[104]

### 5.2. Stem Cell-Derived Exosomes

The current study of stem cell-derived exosomes mostly uses MSCs because of their ex vivo expansion capacity and ethical acceptability [105]. Although MSCs are widely used in regenerative medicine due to their immunosuppressive and anti-inflammatory properties, their dual effects of promoting or inhibiting tumor growth have also been reported depending on the progression of cancer or the type of cancer [106]. It has been suggested that this controversial effect of MSCs in the TME depends on their polarization toward a pro-inflammatory or anti-inflammatory phenotype. In addition, they can polarize the immune system that influence tumor development, and similar to MSCs, MSCs-derived exosomes can also exert both anti- or pro-tumorigenic effects.

For example, exosomes derived from mouse bone marrow MSCs (BM-MSCs) have reported to inhibit angiogenesis in breast cancer (4T1) by downregulating VEGF expression in vivo and in vitro [84]. MiR-16, which is abundant in BM-MSC exosomes, was the source of downregulation of VEGF in the TME of breast cancer. Similarly, a study showed that miR-100-rich human MSCs exosomes reduced the expression of VEGF in breast cancer (MCF-7 and MDA-MB-231) in a dose-dependent manner by modulating mammalian target of rapamycin (mTOR)/hypoxia-inducible factor-1α (HIF-1α) signaling [85]. In addition, the overexpression of miR-124 in human adipose MSCs exosomes has been reported to inhibit ovarian cancer cell proliferation by inducing S-phase arrest through the downregulation of other cyclin-dependent kinases (CDKs) such as CDK2, CDK4, and CDK6 [86]. Despite these reports, there are many findings that MSCs-derived exosomes have a tumorigenic effect. Therefore, more in-depth studies on the relationship between MSCs and cancer are needed for the future safe clinical application of MSCs-derived exosomes for cancer therapy.

### 5.3. Immune Cell-Derived Exosomes

Recent studies have shown that immune cells such as DCs, NK cells, T cells, and macrophages can secrete exosomes that affect many physiological and pathological processes [107]. Thus, immune cell-derived exosomes involved in both immune activation and inhibition have been actively studied as potential therapeutic tools for cancer as well as immune-related diseases.

DCs-derived exosomes (Dexs), the professional antigen-presenting cells necessary to maintain innate and adaptive immunity, have been extensively studied [108]. DCs are involved in both the activation and inhibition of antigen-specific and nonspecific immune responses through the expression of MHC, co-stimulatory, and co-regulatory molecules. Similarly, Dexs are also being studied as therapeutic agents, both applied to immunological activation and inhibition. Compared to DCs, Dexs possess an NK cell lectin-like subfamily K that can activate NK cells on its surface [88] and also express BCL2-associated athanogene 6, which is known to enhance cytokine release from NK cells [89]. One study suggested that TNF in Dexs could kill tumor cells by activating NK cells and inducing the secretion of IFN-γ [87]. Similarly, Basse et al. confirmed that Dexs containing IFN-γ had immunotherapy efficacy by boosting NK cells and T cells in patients with advanced NSCLC [90]. Strategies for loading factors that stimulate a wide range of immune cells into Dexs have also been recently studied. Bone marrow-derived DCs were fed with α-galactosylceramide, which can stimulate IFN-γ producing NK T cells, to enhance antigen-specific T cell responses [91]. Exosomes loaded with OVA and αGC were found to delay tumor progression in a B16-OVA-expressing mouse model. Another group confirmed that OVA or tumor lysate-loaded Dexs enhanced antitumor immunity through TRL3 stimulation during the maturation of bone marrow-derived DCs [92]. Moreover, it has been reported that exosomes derived from DCs expressing hepatocellular carcinoma antigen α-fetoprotein through lentivirus transfection upregulated not only MHC molecules but also co-stimulatory molecules. These exosomes inhibited tumor growth by increasing the number of IFN-γ-producing CD8^+^ T cells, increasing IL-2 expression, and decreasing regulatory T cells [93].

Similar to Dexs, NK-derived exosomes are also highly promising antitumor therapeutic candidates. NK-derived exosomes are known to contain a typical NK marker such as CD56 and cytotoxic molecules including granulysin, granzyme, perforin, and Fas ligand [96]. One of the unique features of human NK cells is that they secrete lytic granules upon activation. In particular, these lytic granules essentially use their cytotoxic molecules to activate their cellular cytotoxicity [109,110,111]. One study suggested that the Fas ligand and perforin of NK-derived exosomes increase the proliferation rate of NK cells in vivo and exert antitumor effects in melanoma mouse xenograft models through NK-mediated cytotoxicity [94]. Interestingly, the glioblastoma mouse model was treated with dextran sulfate to block scavenger receptor A, thereby preventing the ingestion of NK exosomes in the liver, resulting in a significant accumulation of exosomes in the tumors [95]. In addition, a method for the large-scale isolation of exosomes derived from activated NK cells through ex vivo expansion culture has been developed, and it has been confirmed to exhibit toxic activity on several cancer cell lines such as acute lymphoblastic leukemia (SupB15, NALM-6), neuroblastoma (CHLA-136, CHLA-255), and breast cancer (MCF7) [96].

While DC-derived exosomes have been extensively studied, there are few studies on exosomes derived from T cells. T cell-derived exosomes, similar to their parental cells, are mainly associated with antiviral and antitumor responses, and they carry the TCR/CD3 complex from activated T cells [64]. One study reported that exosomes derived from CD8^+^ T cells can induce the apoptosis of mesenchymal stem cells in tumor-bearing mice, thereby attenuating tumor growth [97]. Chimeric antigen receptor engineered T (CAR-T) cells provide a new strategic method for cancer immunotherapy. The target specificity of CAR-T cells is determined by an antibody-derived single-chain variable fragment in the CAR structure. CAR-T cells show poor therapeutic effects in solid tumors due to stroma-rich matrixes [112]. Thus, cell-free exosomes, which are small nanometers in size, may be able to penetrate the site of the tumor, targeting specific antigens and attacking tumor cells [113].

Macrophage-derived exosomes can also regulate the immune response and induce macrophage polarization. From the results of analyzing the mRNA content of activated macrophages and exosomes derived from these cells, it can be inferred that the state of macrophages was reflected in the secreted exosomes [114]. One study confirmed that exosomes derived from M1 polarized macrophages could be used as immunopotentiators for cancer vaccines [98]. In addition, Paclitaxel-loaded macrophage-derived exosomes overcame multidrug resistance in MDCKMDR1 (Pgp+) cells, resulting in a 50-fold increase in cytotoxicity [49]. Overall, with a deep understanding of the role of immune cell-derived exosomes in cancer and advances in exosome engineering that can enhance the efficacy and targeting ability of exosomes, the potential of immune cell-derived exosomes in cancer therapy is very promising.

### 5.4. Other Cells-Derived Exosomes

In addition to the above-mentioned cells, therapeutic applications using exosomes derived from various cells have been studied. The HEK 293T cell line is often used to deliver exogenous therapeutic proteins, nucleic acids, and drugs. For example, HEK293T cells-derived exosomes-loaded super-repressor IκB inhibits the inflammatory responses by reducing the secretion of the NF-κB-mediated pro-inflammatory cytokines such as TNF-α, IL-1β, and IL-6 contributing to cancer development [99]. In another study, HEK293T-derived exosomes that harbor native PH20 hyaluronidase were reported. The engineered exosomes were able to penetrate deeply into the tumor foci via the degradation of hyaluronic acids in the extracellular matrix, resulting in tumor growth inhibition and an increased infiltration of T cells into the tumor [100].

Furthermore, another study identified that the encapsulation of doxorubicin in HEK293T-drived exosomes has potential properties for the effective delivery into target cells. The use of engineering exosomes-loaded doxorubicin via electroporation enhances the cellular uptake efficiency and the therapeutic effect compared to other doxorubicin formulations [101]. In the NSCLC model, exosomes derived from HEK293T cells also mediated the transfer of miRNA-497, which significantly suppressed cell proliferation, migration, and the angiogenesis of tumors. The miRNA loaded in exosomes is more stable than the free miRNA and has a longer cycle time, which is advantageous in controlling cancer development [102].

Recently, adipocyte-derived exosomes that are involved in the response and regulation of metabolic states are emerging as promising therapeutic agents. One study reported that 3T3-L1 adipocyte-derived exosomes could enhance the expression of MMP3 in cancer cells that alleviated lung cancer metastasis by activating MMP9 [103]. On the other hand, Lazar et al. observed the role of adipocyte-derived exosomes that are involved in tumor progression and invasion [104].

## 6. Challenges and Perspectives

The outstanding advantage of exosomes as a drug delivery vehicle lies in their biological origin, which is related to their biocompatibility as a natural nanoplatform. In addition, exosomes have excellent performance in prolonging blood circulation time, increasing tumor targeting, and tumor inhibition. Notably, the use of autologous exosomes has suggested a promising therapy as a personalized nanomedicine [90]. However, therapeutic applications of exosomes have encountered critical challenges for clinical use.

### 6.1. Large-Scale Production for the Therapeutic Use of Exosomes

One of the major challenges to realizing exosomes-based treatments is the low productivity of exosomes. The methods for exosome isolation from the cultured medium are based on ultracentrifuge or filtration at laboratory scales. The yield of exosomes obtained from these methods may vary slightly depending on the type of donor cells but is generally too low. The yield of exosomes is typically less than 1 µg of exosomal protein per 1 mL of culture medium, while the functional dose of exosomes is approximately 10–100 µg exosomal protein per mouse in most studies [115,116,117]. The exosome-containing medium is generally prepared by culturing exosome-producing cells over several days and varies greatly depending on the type of producing cells, but the number of exosomes in the culture medium reaches the upper limit after about 14 h of incubation [118]. Thus, bioreactors can be useful for improving the yield of exosomes. One study found that using a hollow fiber bioreactor can increase the yield of exosomes by 5–10 times [119]. However, since the samples obtained also contain larger vesicles (200–800 nm in diameter), it is not clear whether the yield of exosomes actually increased using a bioreactor. Some studies showed that exosome production was enhanced by providing exosome producing cells with stressful environments such as hypoxia, low pH, and anti-cancer drugs [120,121,122]. However, the therapeutic effectiveness and safety of exosomes secreted from stressed cells should be carefully evaluated, as cellular stress can alter the composition of exosome content and adversely affect the recipient cells [120]. Additionally, it is likely to overestimate the amount of exosome due to contaminants derived from dead cells such as apoptosis bodies. Therefore, technologies and strategies must be developed to produce quality-controllable exosomes on a large scale and to rapidly purify exosomes.

### 6.2. The Heterogeneity of Exosomes

Recent reports suggest that exosomes derived from the same parent cell may have different molecular composition [3,123,124]. In addition, tumor cell-derived exosomes were able to target the donor tumor but exhibited a potential risk of tumorigenesis or improved tumor growth. The heterogeneity of exosomes can also arise due to the difficulty of distinguishing all extracellular vesicles (exosomes, microvesicles, and apoptotic bodies) that share similar properties such as size and density [1,125,126]. Therefore, it may be necessary to discover key components that cause therapeutic or side effects for the therapeutic application of exosomes. There are also unmet needs to establish new standards for reporting research on traits such as exosome diversity, content, and origin. Such standards will be essential for incorporating exosomes into clinical use. Indeed, in recent years, many researchers have been working on the analysis of single vesicles for the clinical application of exosomes [127,128,129]. Ultimately, a better understanding of the heterogeneity and molecular composition of EVs could allow us to determine which subpopulations are better suited for specific exosome-based therapeutics.

In terms of engineering, therapeutic molecules including chemical drugs, nucleic acids, and proteins have been loaded into exosomes. In the process of the engineering, exosomes and donor cells may be affected on exosome content or protein composition. Especially, engineering using physical or chemical stimulation may induce changes in the size and surface potential of the exosome. This may affect the bioactivity and therapeutic efficacy of the exosome. Therefore, exosome engineering should be developed with little impact on the bioactivity, morphology, and composition of exosomes. In addition, similar with content analysis, the developments need to be linked to standards for the evaluation of encapsulation methods.

Despite the challenges, the exosomes have shown a great potential in clinical trials for cancer therapy (Table 3) and would be the next generation of nanoplatforms for advanced therapeutic applications.

## Figures and Tables

**Table 1:** Studies for the loading of therapeutic molecules into exosome.

Method	Drug Loaded	Advantages	Disadvantages	References
Passive Loading Methods				
Incubation with Drugs	Small hydrophobic molecules (curcumin and doxorubicin)	Simplest method Increasing solubility and stability of the hydrophobic drugs in blood circulation	Low drug-loading efficiency Not efficient for large molecules	[42,43]
Active Loading Methods				
Electroporation	Chemotherapeutic drug (doxorubicin and paclitaxel)	Loading with large molecules possible Applicable for nucleic acids	Low drug-loading efficiency (hydrophobic drugs) Cargo aggregation	[45,63,64]
Sonication	Chemotherapeutic drug (doxorubicin and paclitaxel), small RNAs	High drug-loading efficiency Applicable for nucleic acids	Deformation of membrane Low drug-loading efficiency (hydrophobic drugs)	[49,65]
Extrusion	Catalase	High drug-loading efficiency	Deformation of membrane Limitation of membrane	[50]
Freeze and Thaw Cycles	Proteins and peptides	Fusion of membranes possible	Low drug-loading efficiency Exosome aggregation	[50,66]
Click Chemistry	Drugs and nucleic acids	Quick and efficient reactions High specificity	Impairing the functionality of surface proteins	[67]
Exosome-mimic				
Mimetic Nanovesicles	Chemotherapeutic drug (doxorubicin and paclitaxel)	Easier to manufacture High the therapeutic delivery efficiency High yield production	Require to understand cargo loading (cellular uptake, cargo release, and fate of vesicles)	[68,69,70,71]

**Table 3:** Human clinical trials of exosomes in cancer therapy.

Cancer Type	Phase	Source of Exosomes	Results and Status	References
Melanoma	Phase I (*n* = 15)	Immature dendritic cells pulsed with MAGE 3 tumor peptides	Active, Not recruiting	[130]
Non-small cell lung cancer	Phase I (*n* = 4)	Immature dendritic cells pulsed with MAGE-A3, -A4, -A10, and MAGE-3DPO4 tumor peptide	Recruiting	[131]
Non-small cell lung cancer	Phase II (*n* = 22)	IFN-γ- matured dendritic cells pulsed with MAGE-A1, -A3, NY-ESO-1, Melan-A/MART1, MAGE-A3-DP04, EBV tumor peptides	Recruiting	[90] NCT01159288
Colon cancer	Phase I (*n* = 40)	Autologous ascites combined with GM-CSF	Active, Not recruiting	[132]
Colon cancer	Phase I (*n* = 35)	Plant loaded with curcumin	Active, Not recruiting	NCT01294072
Pancreatic cancer	Phase I (*n* = 28)	Mesenchymal stem cells	Not yet recruiting	NCT03608631

## Abbreviations

EVs	Extracellular Vesicles
ILVs	Intraluminal Vesicles
ESCRT	Endosomal Sorting Complexes Required for Transport
MVBs	Multivesicular Bodies
MHC II	Myosin Heavy Chain Class II Proteins
miRNA	MicroRNA
mRNA	Messenger RNA
IFN-γ	Interferon γ
TEXs	Tumor-derived exosomes
NK	Natural-Killer
TME	Tumor Microenvironment
NSCLC	Non-Small Cell Lung Cancer
HGF	Hepatocyte Growth Factor
HEK293T	Human Embryonic Kidney 293T
OVA	Ovalbumin
IL3	Interleukin 3
SIRPα	Signal-Regulatory Protein Alpha
DCs	Dendritic Cells
MSCs	Mesenchymal Stem Cells
Dexs	Exosomes Derived from DCs
